# From Reflexes to Prediction: Kathleen E. Cullen’s Contribution to Modern Vestibular Neuroscience and Clinical Otoneurology—A Conceptual Narrative Review

**DOI:** 10.3390/audiolres16040096

**Published:** 2026-06-28

**Authors:** Leonardo Manzari

**Affiliations:** MSA ENT Academy Center, Via Tommaso Piano 16, 03043 Cassino, Italy; lmanzari1962@gmail.com; Tel.: +39-338-2864625

**Keywords:** vestibular system, self-motion, neural encoding, behavioral context, vestibular cerebellum, internal models, reafference, multisensory integration, velocity storage, visual dependence

## Abstract

**Background**: The vestibular system has traditionally been interpreted within a reflex-based framework, mainly centered on gaze stabilization, vestibulo-ocular reflex pathways, and peripheral vestibular deficits. This model remains essential, but it is insufficient to explain the full spectrum of postural, perceptual, visual-motion, and self-motion complaints observed in contemporary clinical otoneurology. **Objective**: This conceptual narrative review examines selected representative works by Kathleen E. Cullen as landmarks in a broader transition from reflex physiology to predictive, multimodal, context-dependent, body-centered self-motion control. **Methods**: This is not a systematic or bibliometric review. Papers were selected because they mark distinct conceptual steps in Cullen’s work: neural encoding of self-motion, peripheral and central coding strategies, multimodal integration, active versus passive self-motion, reafference suppression, body-centered encoding, proprioceptive prediction, vestibular cerebellar internal models, sensory reweighting, and clinical translation. **Synthesis**: Angelaki and Cullen’s 2008 synthesis and Cullen’s subsequent work demonstrate that vestibular processing is inherently multimodal from the earliest central stages and that neural representations of self-motion depend on behavioral context. Vestibular nuclei, visual-vestibular networks, and vestibular cerebellar circuits integrate labyrinthine signals with optic flow, proprioceptive, oculomotor, motor, cerebellar, cortical, and contextual information. This architecture enables the brain to distinguish expected from unexpected motion, suppress predictable vestibular reafference during voluntary action, compute internal estimates of body motion, adapt to altered sensory reliability, and reweight sensory inputs according to task demands. **Conclusions**: The clinical relevance of this trajectory is substantial. Patients may show preserved high-acceleration vestibulo-ocular reflex responses while experiencing persistent instability, visually induced dizziness, defective self-motion perception, or abnormal sustained vestibular processing. Such dissociations are not paradoxical when the vestibular system is understood as a predictive, distributed, body-centered control system. Cullen’s long lesson offers a neurophysiological foundation for a modern vestibular grammar in which clinical findings are interpreted across the reflexive, perceptual, postural, visual-vestibular, sustained, and predictive domains.

## 1. Introduction

The vestibular system has long occupied a privileged position in neuroscience because of the apparent clarity of its reflex pathways. The semicircular canals and otolith organs detect angular and linear head motion, vestibular afferents transmit this information to the vestibular nuclei, and short-latency motor pathways stabilize gaze and posture. This architecture has provided one of the most elegant models of sensorimotor transformation in the nervous system. In clinical otoneurology, it has been extraordinarily productive: it has allowed physicians to diagnose peripheral vestibular loss, quantify vestibulo-ocular reflex function, and interpret several acute vestibular syndromes with remarkable precision.

Yet this framework is incomplete. Patients do not experience the vestibular system as an isolated reflex arc. They experience it as the ability to stand, walk, turn, orient, anticipate, tolerate visual motion, recover after perturbation, and maintain a stable sense of self-motion in space. A patient with dizziness does not complain of an abnormal gain value; he or she complains of a body that no longer feels reliable in the world. This difference between what is measured by a single reflex test and what is lived by the patient represents one of the major challenges of contemporary vestibular medicine.

The scientific work of Kathleen E. Cullen offers a powerful framework for addressing this challenge. Across experimental, computational, and review contributions, Cullen has progressively reframed the vestibular system as an active, multimodal, predictive, and body-centered system for self-motion control. Her work demonstrates that vestibular pathways are not passive relays of labyrinthine information. Instead, they integrate vestibular signals with proprioceptive, oculomotor, motor, cerebellar, cortical, and contextual information to distinguish expected from unexpected motion, active from passive movement, head motion from body motion, and sensory inflow from motor prediction [1].

A decisive early formulation of this transition was the 2008 Annual Review of Neuroscience article by Angelaki and Cullen, which described the vestibular system as a multimodal sense in which peripheral head-motion signals are immediately embedded in computations involving gravity, proprioception, vision, corollary discharge, reference-frame transformations, navigation, spatial orientation, and behavioral goals [1]. In retrospect, this review reads as a blueprint for the clinical shift from reflex testing toward predictive self-motion control.

This review uses Cullen’s work not as a biographical subject, but as a conceptual route through modern vestibular neuroscience. The purpose is scientific and translational: to show how experimental physiology provides a neurophysiological foundation for clinical otoneurology in which dissociations between tests, symptoms, posture, visual dependence, and self-motion perception are no longer exceptions but expected expressions of a distributed vestibular system.

The central thesis is that Cullen’s long lesson is not simply that the vestibular system detects motion. It is that the vestibular system interprets self-motion in relation to action, prediction, body configuration, gravity, and behavioral context. This lesson is essential not only for basic neuroscience, but also for clinicians facing patients whose symptoms cannot be reduced to a damaged peripheral sensor or to a single abnormal reflex.

## 2. Approach and Selection of Representative Papers

This manuscript is designed as a conceptual narrative review. It is not intended to be a systematic review, a bibliometric analysis, or an exhaustive reconstruction of the complete scientific production of Kathleen E. Cullen. The selection of papers is intentionally limited and concept driven. The selected works are treated as conceptual landmarks that allow the reader to follow a trajectory in vestibular neuroscience: from reflex-based processing to predictive, multimodal, body-centered control of self-motion.

The included papers were chosen because each addresses a distinct computational or translational step. Angelaki and Cullen’s 2008 synthesis is included as an early blueprint for a multimodal vestibular sense, integrating canal-otolith computation, gravity, proprioception, corollary discharge, reference frames, navigation, and spatial orientation [1]. Brooks and Cullen’s 2009 work provides evidence for body-motion estimates in the rostral fastigial nucleus [2]. The 2011 review, The Neural Encoding of Self-Motion, serves as a conceptual gateway because it explicitly frames self-motion as a multisensory neural construction shaped by vestibular, visual, proprioceptive, and motor efference cues and by behavioral context [3]. The 2011 review on internal models and vestibular reafference addresses the active/passive distinction and the selective suppression of predictable self-generated vestibular input [4]. The 2012 review in Trends in Neurosciences articulates the wider multimodal architecture of self-motion encoding [5]. The 2015 paper by Carriot, Jamali, and Cullen links internal models and sensory reweighting to adaptation [6]. The 2019 review on vestibular processing during natural self-motion extends this framework toward perception and action in behaviorally relevant contexts [7]. The 2021 review by Cullen and Zobeiri emphasizes proprioception and predictive sensing during voluntary self-motion [8]. Two 2023 contributions expand the framework toward vestibular cerebellar internal models and a broad account of vestibular motor control [9,10].

This selection is therefore not justified by citation counts, chronology alone, or completeness but by conceptual continuity. Together, these papers define coherent scientific grammar: the vestibular system is not merely a peripheral detector feeding reflexes, but a distributed predictive system that integrates motion, body configuration, action, gravity, and context. Foundational theoretical references on reafference and internal models are retained to clarify the historical and computational background of Cullen’s experimental contribution [11,12,13].

## 3. The Multimodal Vestibular Sense: A 2008 Blueprint

Angelaki and Cullen’s 2008 review should be read as one of the earliest comprehensive statements of the transition that now appears clinically unavoidable. The paper begins with the peripheral fact that elegant inner-ear sensors measure head motion but immediately moves beyond that fact: central vestibular processing is described as strongly convergent, multisensory, and multimodal, contributing not only to gaze and posture but also to self-motion perception, navigation, motor coordination, and spatial orientation [1]. The vestibular signal is therefore not treated as a private sensory channel with a single conscious output, but as a distributed computational resource for action and orientation.

Three ideas from that synthesis are particularly relevant for the present review. First, semicircular canal and otolith signals are ambiguous when considered separately, so the brain must combine canal, otolith, and gravity-related information to compute inertial motion, tilt, translation, and orientation in world-centered terms. Second, proprioceptive–vestibular interactions and corollary discharge allow the nervous system to distinguish self-generated from externally imposed head motion through reafference/exafference computations. Third, vestibular information must be transformed across multiple reference frames, including head-centered, body-centered, eye-centered, and world-centered representations, depending on the behavioral problem being solved [1].

For clinical otoneurology, this 2008 article functions as a conceptual hinge. It explains why a patient may appear normal when a single head-centered reflex is tested, yet fail when the nervous system must combine vestibular, visual, proprioceptive, gravitational, and motor-predictive information during real behavior. In that sense, the paper anticipates the core argument of the present manuscript: vestibular disorders should be interpreted not only as lesions of sensors or reflex arcs, but also as disturbances of multimodal self-motion inference.

## 4. The Neural Encoding of Self Motion: A Conceptual Gateway

The 2011 review, The Neural Encoding of Self-Motion, is a pivotal landmark because it places the problem of self-motion at the center of vestibular neuroscience [3]. Cullen emphasizes that vestibular signals alone do not define the perceptual and motor experience of movement. During everyday life, the brain combines labyrinthine input with optic flow, proprioception, and motor efference copy signals to generate neural representations of self-motion. This immediately broadens the clinical question: the patient’s symptom is not simply the output of a canal or otolith sensor, but the result of how the nervous system constructs self-motion from multiple converging signals.

The review also clarifies that the strategy used by the brain to encode self-motion depends on behavioral context. Passive movement, visually guided motion, body-under-head movement, locomotion, and voluntary head turns do not impose the same computational demands. In passive conditions, multimodal cues may be combined through reliability-dependent or weighted integration. During active movement, however, the problem becomes different: the system must distinguish sensory inflow generated by one’s own action from unexpected motion imposed by the external world. Thus, self-motion is not a fixed sensory quantity: it is an inference shaped by task, prediction, reliability, and body configuration.

This point is essential for the clinical argument of the present review. Many bedside and laboratory tests interrogate specific reflex domains under simplified or constrained conditions. Real-life dizziness, however, emerges when self-motion must be inferred in complex contexts: walking while turning the head, navigating optic flow, stabilizing the body on uneven ground, suppressing inappropriate reflex responses, or recalibrating movement after vestibular loss. Cullen’s 2011 synthesis therefore provides the conceptual gateway between experimental neurophysiology and the modern clinic of vestibular dissociations.

## 5. Peripheral Diversity: Calyx, Bouton, Dimorphic Endings, and Parallel Afferent Channels

The transition from reflex physiology to predictive self-motion control should not lead to an abandonment of peripheral vestibular physiology. On the contrary, it requires a more precise reading of the periphery. Vestibular information is already diversified at the sensory periphery. In Cullen’s synthesis, afferents differ not only in discharge regularity, but also in their peripheral endings and hair-cell targets. Bouton endings preferentially contact type II vestibular hair cells and are generally associated with more regular afferents. Calyx endings surround type I hair cells and are typically associated with irregular afferent signaling. Dimorphic afferents, also referred to as D-irregulars, contact both type I and type II vestibular hair cells. Thus, “dimorphic” does not designate a clinical phenotype; it denotes a mixed peripheral innervation pattern linking both hair-cell populations to a single afferent architecture [14,15,16].

This anatomical and physiological heterogeneity gives rise to parallel information channels rather than to a single undifferentiated vestibular output. Irregular afferents generally show higher gains and greater phase leads for rapid or high-frequency components of natural head motion, whereas regular afferents can transmit more information about the detailed time course of motion and show lower detection thresholds across much of the behaviorally relevant range. The distinction is therefore not merely anatomical; it is computational. From the periphery onward, the vestibular system fractionates motion information into channels that differ in dynamics, sensitivity, timing, and coding strategy.

Clinically, this provides a physiological basis for thinking across dynamic domains. High-acceleration tests such as vHIT and rapid perturbation paradigms such as skull vibration-induced nystagmus may preferentially emphasize fast, phasic, transient vestibular signaling. In contrast, caloric, rotational, post-rotatory, optokinetic, VVOR, and VORS paradigms may reveal slower, sustained, or more integrative components of vestibular function. The clinical dissociation between preserved transient reflex responses and persistent body instability therefore begins, at least in part, with the existence of multiple vestibular channels already present at the sensory periphery.

The transient-sustained distinction used clinically should not be reduced to a rigid one-to-one mapping onto irregular and regular afferents. Such a simplification would be physiologically excessive, because vestibular dynamics emerge from the interaction between peripheral afferent properties, end-organ mechanics, central vestibular processing, cerebellar modulation, behavioral context, and task demands. Irregular and regular afferents provide important examples of parallel vestibular information channels, but they do not by themselves explain the full temporal organization of vestibular function [3,14,15,16,17,18].

Nevertheless, Cullen’s work, together with classic and contemporary studies on vestibular afferent diversity, information transmission, and central population encoding, supports the broader principle that vestibular processing is dynamically fractionated from the periphery onward [3,14,17,18]. The vestibular system does not transmit a single, homogeneous motion signal. Rather, it distributes motion information across channels that differ in sensitivity, timing, frequency preference, discharge regularity, information rate, and central weighting [14,17,18]. This principle is clinically more useful than a rigid peripheral dichotomy. It explains why different vestibular tests may reveal different aspects of the same patient’s disorder: a rapid head impulse may probe one dynamic window, whereas caloric stimulation, low-frequency rotation, post-rotatory responses, optokinetic stimulation, VVOR, VORS, or motion-rich behavioral tasks may probe others [5,19,20,21,22]. The same logic has recently been framed clinically as a multiscale acceleration- and jerk-sensing model, in which transient, high-jerk paradigms and sustained, low-frequency paradigms are interpreted as preferential probes of different dynamic activation modes rather than as contradictory measures of a single vestibular output [20].

In this sense, the clinical distinction between transient and sustained vestibular processing should be understood as a functional framework rather than an anatomical label. It does not imply that a given symptom or test result can be assigned to one afferent class alone. Instead, it recognizes that vestibular disorders may selectively affect, spare, or dissociate different temporal and integrative domains of self-motion processing. This provides a safer and more powerful explanation for why patients may show preserved high-acceleration reflexes while remaining symptomatic during sustained motion, visually complex environments, postural tasks, or active self-generated movement. It also prevents the clinician from treating discordant test results as contradictions, when they may instead represent different dynamic windows on a distributed vestibular system.

## 6. Visual-Vestibular Integration and Optic Flow

Optic flow is one of the most powerful extra-vestibular cues for self-motion, and Angelaki and Cullen’s 2008 review already situated visual-vestibular interactions within the broader problem of multimodal spatial orientation [1]. Cullen’s 2011 review places visual-vestibular integration at the center of self-motion encoding, emphasizing that visual cues from retinal image motion can be combined with vestibular signals to estimate heading and movement through space [3]. This principle is also supported by experimental work on visual and vestibular cue integration for heading perception [22]. This is a crucial extension of vestibular physiology. The vestibular system does not operate in darkness as a default state; in everyday life, it is continuously interpreted in relation to visual motion, environmental stability, and contextual expectation.

The visual system can support self-motion perception, but it can also destabilize it. When vestibular reliability is reduced, uncertain, or maladaptively interpreted, the nervous system may overweight visual cues. In some patients, complex visual environments become provocative because optic flow is no longer integrated as one cue among others, but becomes an excessively dominant reference. Supermarkets, traffic, crowds, patterned floors, scrolling screens, or visually busy corridors become clinical stress tests of multisensory inference.

This visual-vestibular dimension is particularly relevant for patients with persistent dizziness, motion sensitivity, or visually induced disequilibrium despite preserved peripheral reflex measurements. In such patients, the symptom does not necessarily indicate that the labyrinth is currently failing. It may indicate that visual motion, vestibular uncertainty, and body-based prediction are being combined abnormally. This is consistent with modern descriptions of persistent postural-perceptual dizziness, in which symptoms are exacerbated by upright posture, movement, and complex visual stimulation [23]. The clinical task is therefore not only to test the canal response, but also to understand whether the patient can correctly integrate or suppress visual motion according to context.

## 7. From Reflex Architecture to Multimodal Self-Motion Encoding

The classical vestibular model begins with sensors and ends with reflexes. The canals and otoliths provide information about head motion; vestibular nuclei transform these signals into motor commands for gaze and postural stabilization; cerebellar circuits calibrate reflex performance. This model remains indispensable. It explains why vestibular loss causes oscillopsia, imbalance, and abnormal responses to head motion and also provides the physiological basis for several clinical tests, including head impulse testing, caloric testing, rotational chair testing, and vestibular-evoked myogenic potentials.

Cullen’s work does not reject this reflex architecture. Rather, it shows that the reflex architecture is embedded in a wider computational system. Vestibular nuclei neurons are not simple relays. They receive direct peripheral vestibular input, but they also integrate proprioceptive, oculomotor, predictive, cerebellar, cortical, and brainstem signals. This multimodal convergence is present at the earliest central stages of vestibular processing and shapes even apparently simple sensorimotor transformations [1,5,10].

The implication is profound. The vestibular system does not operate as a closed circuit between the labyrinth and the eye. It is a component of a larger self-motion network. Its output depends on the behavioral goal: stabilizing gaze, maintaining posture, orienting the body, interpreting movement, or suppressing predicted sensory consequences during voluntary action. A reflex can therefore be preserved while other vestibular domains are impaired. This is one of the reasons why modern clinical otoneurology must move beyond the logic of one test, one lesion, one symptom.

## 8. Active and Passive Self-Motion: Reafference, Exafference, and Internal Models

One of the most important conceptual shifts in Cullen’s work is the distinction between active and passive self-motion. Traditional vestibular experiments often used passive stimulation: the head or body was moved by an external device while neuronal and behavioral responses were recorded. Such paradigms remain essential because they isolate the input–output properties of vestibular pathways. However, most vestibular stimulation in daily life arises from voluntary behavior. We turn our heads, walk, reach, accelerate, brake, and change direction. The sensory consequences of these movements are partly predictable because they are generated by our own motor commands.

The active/passive distinction is not a technical detail of experimental design; it is a conceptual boundary between reflex physiology and predictive self-motion control. Passive motion paradigms ask how vestibular pathways respond when movement is externally imposed. Active motion paradigms ask how the brain interprets vestibular inflow when movement is self-generated, predictable, accompanied by proprioceptive feedback, and embedded in an ongoing behavioral act. The question therefore shifts from ‘how much does the vestibular system respond when the head is moved?’ to ‘what does the brain do when that movement is produced by the subject, predicted by the motor command, and interpreted within a moving body?’.

This distinction is often underestimated. Cullen does not deny the importance of the reflex. Rather, she places it within its proper functional context. The VOR remains fundamental for gaze stabilization, and passive reflex testing remains indispensable in clinical practice. However, the VOR does not exhaust vestibular function. The real vestibular system operates inside a body that acts, predicts, receives proprioceptive feedback, interacts with visual motion, and must continuously distinguish expected from unexpected sensory consequences.

Clinically, this point is decisive. A vestibular system that responds normally to passive head impulses may still fail when self-motion must be predicted, filtered, and integrated during active behavior. The patient may not be symptomatic because the labyrinth cannot generate a reflex response, but because the central vestibular system cannot correctly interpret the meaning of that response in relation to action, posture, vision, proprioception, and context. This is the conceptual doorway through which Cullen’s experimental physiology becomes modern clinical otoneurology.

The central clinical problem in many such patients is not the absence of a vestibulo-ocular reflex, but the failure to build a stable prediction of self-motion in the world. In daily life, most vestibular stimulation is actively generated rather than passively imposed. The brain therefore does not merely ask whether the vestibular signal is present or absent; it asks whether incoming vestibular, proprioceptive, visual, and motor signals match the predicted sensory consequences of action. When prediction and sensory inflow match, self-motion is experienced as coherent and stable. When they do not, prediction error emerges, and the patient may experience dizziness, instability, or loss of confidence in movement despite preserved reflex responses. This interpretation is consistent with Cullen’s work on vestibular reafference, natural self-motion, proprioceptive prediction, and internal models of self-motion [4,7,8,9].

The nervous system must therefore distinguish reafference, the sensory inflow caused by one’s own action, from exafference, the sensory inflow caused by unexpected events in the external world. This distinction is critical for stability. If all self-generated vestibular signals automatically triggered full postural reflexes, voluntary movement would be counteracted by the very reflexes designed to protect balance. Conversely, if unexpected vestibular signals were ignored, the organism would fail to respond to slips, trips, pushes, or sudden perturbations. This principle was explicitly framed in the 2008 review as a central operation of vestibular multimodal processing [1].

Cullen and colleagues showed that primary vestibular afferents do not substantially distinguish active from passive movement [4]. The sensory periphery faithfully transmits motion information. The crucial distinction emerges centrally. A specific population of vestibular nuclei neurons, historically termed vestibular-only neurons, shows marked attenuation during active self-generated head movement while continuing to respond to passive or unexpected components of motion. In contrast, neurons related to gaze stabilization can remain robust when the behavioral goal is to stabilize gaze. The vestibular system therefore does not apply a global suppression rule but applies a task-dependent computational strategy.

This selective suppression is consistent with an internal model of the sensory consequences of action. An efference copy of the motor command is used to predict the sensory feedback expected from voluntary movement. When proprioceptive feedback and motor expectation match, a cancellation signal can attenuate predictable vestibular reafference. When the motion is unexpected or mismatched, the signal remains available for postural and perceptual computation. In this framework, the vestibular system is not simply measuring motion; it is evaluating whether the motion was expected, self-generated, or externally imposed.

Importantly, this active/passive distinction should not be misread as a therapeutic recommendation to privilege passive movement over active rehabilitation. It is a computational distinction, not a prescriptive opposition. Passive motion paradigms reveal how vestibular pathways respond to externally imposed perturbations; active motion paradigms reveal how the brain predicts, filters, and interprets sensory inflow generated by the subject’s own action. Therefore, a Cullen-inspired clinical translation does not reduce rehabilitation to passive exposure. Rather, it highlights the need to progressively restore the patient’s ability to generate, predict, tolerate, and recalibrate active self-motion in real-world contexts.

## 9. From Head Motion to Body Motion

Clinical vestibular testing frequently measures head-centered responses. The vHIT evaluates high-acceleration canal-driven gaze stabilization. Caloric and rotational tests interrogate horizontal canal pathways at different frequency and time scales. These tests are essential, but patients rarely describe their illness in head-centered language. They describe instability of the body, uncertainty of posture, difficulty walking in darkness, intolerance of complex visual scenes, or a loss of confidence in movement.

Brooks and Cullen’s work on the rostral fastigial nucleus provides a neurophysiological bridge between these worlds [2]. Their findings indicate that neurons in this cerebellar region can explicitly encode body movement in space by integrating vestibular and proprioceptive inputs. Some neurons behave like head-motion neurons, primarily reflecting vestibular input. Others combine vestibular and neck proprioceptive information to estimate body motion. This is a decisive transition from head-centered vestibular coding to body-centered self-motion estimation.

The clinical implication is direct. A patient may show preserved head impulse gains and still experience body-based instability if the problem lies in the transformation of head motion into a body-referenced estimate, in the integration of vestibular and proprioceptive signals, or in the use of these estimates for posture and orientation. A normal reflex measurement therefore cannot be equated with a normal vestibular experience. The patient’s complaint may arise downstream from the reflex, in the computations that translate head-based sensory information into body stability in the world.

## 10. Proprioception and Predictive Sensing During Natural Behavior

Proprioception is not an accessory to vestibular function; it is a partner in self-motion control. During natural behavior, vestibular sensors encode head motion in space, while proprioceptors encode the configuration and movement of the neck, trunk, and limbs. The brain must combine these signals to know whether the head moved on the body, whether the body moved under the head, whether the whole body moved in space, or whether an external perturbation occurred.

Cullen and Zobeiri emphasized that vestibular and proprioceptive systems cooperate to generate a predictive sense of active self-motion [8]. This is particularly important because voluntary movement changes the meaning of vestibular inflow. A given head acceleration is not interpreted in isolation; it is interpreted in relation to intended action, proprioceptive feedback, and behavioral context. The vestibular system is therefore part of a sixth-sense architecture in which movement is sensed, predicted, and interpreted through the body.

This has major clinical consequences. Many patients perform adequately during simplified passive tests but fail during real-life movement. Walking in a supermarket, turning in a crowd, descending stairs, moving the head while navigating uneven terrain, or performing a dual task imposes a complex combination of vestibular, visual, proprioceptive, and predictive demands. If clinical reasoning remains limited to passive reflex testing, these impairments may appear disproportionate or unexplained. A predictive proprioceptive-vestibular framework makes them physiologically intelligible.

## 11. Vestibular Cerebellum, Gravity, and Sustained Self-Motion

The vestibular cerebellum is central to the transition from reflex physiology to predictive self-motion control. The floccular lobe contributes to gaze stabilization and vestibulo-ocular reflex calibration. The anterior vermis participates in transforming vestibular signals into body-centered reference frames and in predicting the sensory consequences of active motion. The nodulus and ventral uvula contribute to the internal representation of spatial orientation and self-motion relative to gravity.

Cullen’s 2023 review on internal models of self-motion synthesizes these functions into a broader computational view [9]. The vestibular cerebellum integrates vestibular input with visual, proprioceptive, motor, and contextual signals to build internal models of eye, head, body movement, and orientation relative to gravity. These models are updated when environmental conditions, biomechanics, or sensory reliability change. They allow the system to maintain stability not by reflex alone, but by prediction and adaptive calibration. This view is coherent with broader evidence that the brain uses internal models to estimate gravity and linear acceleration [13].

This cerebellar framework is clinically important for persistent dizziness and sustained vestibular processing. Symptoms may persist not because a peripheral canal reflex remains grossly abnormal, but because the system that estimates orientation, gravity, body motion, or ongoing self-motion remains maladapted. The nodulus and uvula, in particular, provide a conceptual bridge to velocity storage, verticality, gravitational orientation, visual dependence, and prolonged motion sensitivity. In clinical terms, the sustained component of vestibular processing may be as important as the transient reflex response, and its interpretation should be connected to classical models of velocity storage as a central temporal integrator [21].

This bridge between velocity storage, gravity-dependent orientation, active/passive processing, and conflict detection has been explicitly developed in the broader velocity-storage literature. Lackner and DiZio emphasized that velocity storage contributes not only to the persistence of vestibulo-ocular responses, but also to spatial orientation, disorientation, and gravity-dependent motion sickness [24]. In the same review, they highlighted Cullen’s work on differential processing of active versus passive canal and otolith stimulation, including the functional partitioning of vestibular nuclei neurons into vestibular-only, position-vestibular-pause, and floccular target neurons. They also noted that reciprocal fastigial-vestibular connectivity may provide a circuit-level substrate for detecting conflict between the predicted and actual sensory consequences of motion, a concept further articulated by Oman and Cullen [25]. For the present clinical framework, this connection is crucial: velocity storage should not be viewed only as a generator of prolonged nystagmus, but as part of a wider system in which gravity, body orientation, sensory conflict, internal models, and action-dependent prediction converge.

## 12. Adaptation, Sensory Reweighting, and Compensation

Compensation is often described as recovery after vestibular loss, but Cullen’s work invites a broader view. Compensation is not merely the restoration of reflex gain. It is a dynamic reorganization of prediction, sensory reliability, and behavioral strategy. When vestibular input becomes unreliable or violates expectation, the nervous system updates internal models and changes the relative weight assigned to vestibular, visual, proprioceptive, and somatosensory cues.

Carriot, Jamali, and Cullen connected these processes to rapid adaptation in vestibular pathways [6]. Their discussion of altered gravity and spaceflight illustrates the principle with particular clarity. When the sensory consequences of movement no longer match the brain’s earth-based expectations, the system initially experiences conflict, disorientation, and impaired sensorimotor performance. Adaptation requires both updating the internal model and reweighting alternative sensory references.

The same logic applies clinically. After acute vestibular loss, the brain may compensate by increasing reliance on vision or proprioception. This can be adaptive in the short-term but maladaptive in the long-term if visual dependence becomes excessive or if the patient avoids movement. Persistent dizziness may therefore arise not only from an abnormal sensor, but from maladaptive weighting of available sensory references. Rehabilitation in this framework is not generic exercise: it is guided exposure designed to recalibrate prediction, reduce inappropriate sensory dependence, and restore confidence in body-centered self-motion.

## 13. Clinical Translation: Dissociations as Physiological Clues

The original clinical contribution of this review is to translate Cullen’s experimental physiology into a practical way of reading vestibular dissociations. Preserved high-acceleration canal responses on vHIT may coexist with postural instability, visually induced dizziness, motion sensitivity, impaired spatial orientation, or abnormal sustained responses. Within a predictive and distributed vestibular framework, such findings are not contradictions; they indicate that different domains of the system have been interrogated.

In this grammar, vHIT remains indispensable for evaluating rapid, high-acceleration, head-centered vestibulo-ocular reflex function, especially in acute vestibular syndromes and bilateral vestibular loss. However, normal vHIT gains do not exclude abnormalities in self-motion estimation, multisensory integration, vestibular cerebellar computation, or sensory reweighting. HIMP/SHIMP, VVOR/VORS, optokinetic responses, rotational chair testing, SVIN, VEMPs, subjective verticality, and postural observation should therefore be interpreted as complementary windows rather than competing explanations.

Visual-vestibular and sustained temporal tests are particularly important in patients whose symptoms are provoked by optic flow, crowds, scrolling environments, or prolonged motion. In these cases, the problem may involve abnormal weighting of visual cues, impaired suppression or integration of vestibular input, or disturbed velocity-storage dynamics rather than a simple failure of the peripheral reflex. Low-frequency rotation, post-rotatory responses, and time constants address temporal integration and may reveal abnormalities invisible to brief impulse testing [21].

Thus, the clinician’s task is not to decide which single test is true, but to identify which functional domain each test probes and how the resulting pattern explains the patient’s real-life symptoms. Dissociation becomes information.

## 14. Functional Clinical Phenotypes Suggested by the Framework

For clinical purposes, the predictive self-motion framework may be translated into functional phenotypes. These are not diagnostic categories, but interpretive patterns that organize symptoms and test results across vestibular domains.

A reflex-preserved/body-unstable phenotype refers to patients with preserved high-acceleration reflexes but persistent imbalance or poor confidence during real-life movement, suggesting impaired body-centered estimation or postural use of vestibular information. A visual-motion sensitive phenotype is dominated by symptoms in optic-flow-rich environments, implying abnormal visual weighting within self-motion inference. A sustained self-motion or velocity-storage phenotype emerges when difficulty is greater during low-frequency rotation, post-rotatory behavior, prolonged motion sensitivity, or delayed recovery after movement than during brief impulses.

A predictive mismatch phenotype describes patients who may appear relatively stable during simplified passive testing but become symptomatic during active movement, when efference copy, proprioceptive matching, and reafference cancellation are required. Finally, a maladaptive compensation or sensory-reweighting phenotype identifies patients who remain excessively dependent on visual or somatosensory references after vestibular injury. These patterns are proposed as hypothesis-generating clinical tools and should be prospectively validated.

## 15. A Domain-Based Clinical Framework

The proposed clinical framework is domain-based. Reflexive function asks whether rapid vestibulo-ocular reflex pathways are intact. Perceptual function asks whether self-motion and orientation are stable. Postural function asks whether vestibular information can be used for body stability under real-world constraints. Visual-vestibular function asks whether optic flow and visual context are integrated or suppressed appropriately. Sustained function asks whether vestibular signals are temporally integrated across low-frequency and post-rotatory domains. Predictive function asks whether the brain correctly distinguishes expected from unexpected motion and flexibly weights available sensory references.

This approach does not diminish classical vestibular tests; it increases their interpretive value by assigning each test to a functional domain. A normal vHIT indicates preservation of one rapid reflex window, not global normality. A weak sustained rotational response need not contradict a normal impulse response; it may reveal a different temporal domain. A visually dependent patient may not have a new peripheral lesion, but a maladaptive weighting strategy. The aim is therefore to read the patient across domains rather than to force all findings into a single-test explanation.

## 16. Implications for Vestibular Rehabilitation

If vestibular dysfunction is understood as impaired predictive self-motion control, rehabilitation should be planned as domain-specific recalibration rather than as generic symptom provocation. Its goals are to restore reliable self-motion estimation, reduce maladaptive sensory dependence, rebuild prediction, and recover confidence in body-centered movement, consistent with contemporary concepts of vestibular compensation and rehabilitation [26].

Therapy may therefore target different domains according to the patient’s pattern: gaze stabilization and dynamic visual targets in the reflexive domain; graded optic-flow exposure and visual-motion tolerance in the visual-vestibular domain; stance, gait, turns, uneven surfaces, darkness, and dual tasks in the postural/body-centered domain; active movement, anticipation, and controlled mismatch in the predictive/adaptive domain; and graded rotational or motion exposure in patients dominated by sustained or velocity-storage-like symptoms.

The active/passive distinction should not be translated into a simplistic therapeutic opposition. Passive perturbations can probe detection and responses to unexpected motion, but daily recovery requires active, intentional, context-rich movement. Only active self-motion fully engages efference copy, proprioceptive matching, reafference cancellation, visual-vestibular context, and body-centered estimation [4,8,24]. Rehabilitation should therefore be prescribed according to the dissociation pattern, asking not only which sensor is abnormal, but which prediction, sensory weight, body estimate, or behavioral context remains unstable.

## 17. Limitations of the Present Conceptual Review

This manuscript is intentionally conceptual. It does not provide a systematic search strategy, quantitative synthesis, or bibliometric ranking of Cullen’s scientific production. The selected papers are representative rather than exhaustive. This design is appropriate for reconstructing a conceptual trajectory, but it limits claims about the totality of the literature.

Another limitation is translational. The clinical phenotypes and domain-based interpretations proposed here are derived from experimental physiology and clinical reasoning. They should be regarded as a framework for interpretation and hypothesis generation, not as validated diagnostic categories. Future work should test whether specific patterns of vHIT, SHIMP, VVOR, VORS, optokinetic responses, rotational chair behavior, postural metrics, and patient-reported symptoms can be reliably mapped onto the proposed domains.

Finally, the manuscript is author-centered. This was a deliberate choice, because Cullen’s work offers a coherent route through the transition from reflex physiology to predictive self-motion control. However, modern vestibular neuroscience is a collective field. The present review should therefore be read as one conceptual pathway into the broader literature, not as an attempt to reduce the field to a single investigator.

## 18. Proposed Clinical Implications

- Preserved high-acceleration vestibulo-ocular reflex responses should not be used as proof that vestibular function is globally normal. They indicate that one reflex domain is preserved.
- Test dissociations should be interpreted as physiological clues. Differences between vHIT, SHIMP, VVOR, VORS, optokinetic responses, SVIN, caloric responses, and rotational chair findings may reveal the distributed organization of vestibular processing.
- Chronic dizziness should be evaluated not only as residual peripheral damage but also as impaired prediction, maladaptive sensory reweighting, abnormal visual dependence, or disturbed sustained self-motion integration.
- In predictive terms, the key question is whether the patient can still generate a coherent and trusted estimate of self-motion during active, visually, and proprioceptively complex behavior.
- Vestibular rehabilitation should be designed by domains. The goal is not simply to improve a gain value, but to restore reliable self-motion estimation, reduce maladaptive sensory dependence, recalibrate prediction, and rebuild confidence in movement.
- A modern vestibular assessment should ask how the patient uses vestibular information in action, vision, posture, gravity, and time, not only whether a single reflex is measurable. This approach does not replace peripheral diagnosis; it completes it by placing each reflex measure within a broader self-motion framework.

## 19. Conceptual Figure

**Figure 1:**
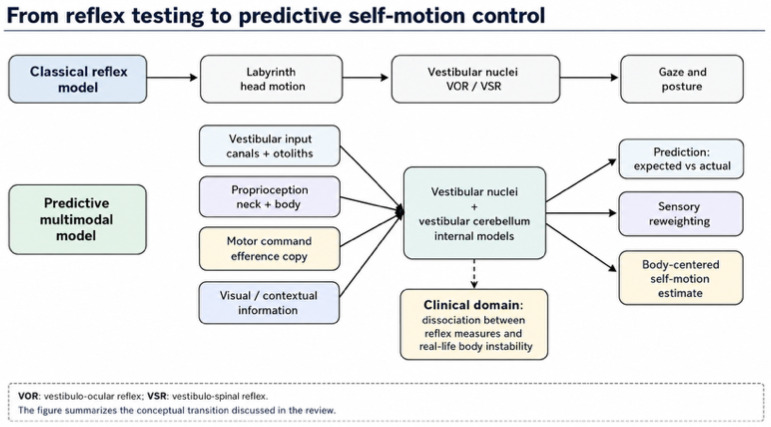
From reflex vestibular testing to predictive self-motion control. The upper pathway represents the classical reflex model, in which labyrinthine head-motion signals drive vestibular nuclei and short-latency gaze/postural reflexes. The lower pathway represents a predictive multimodal model in which vestibular input is integrated with proprioception, visual context, motor commands, and cerebellar internal models to compute expected versus actual motion, sensory reweighting, and body-centered self-motion estimates. The dashed arrow indicates the clinical transition from preserved head-based reflexes to real-life body-based instability, highlighting the dissociation between reflex integrity and impaired predictive self-motion control.

The conceptual transition proposed in this review is summarized in Figure 1. The figure illustrates the shift from a classical reflex-centered interpretation of vestibular function toward a predictive, multimodal, and body-centered model of self-motion control.

## 20. Foundational Papers and Conceptual Transition Abbreviations

The main conceptual steps discussed in this review are summarized in Table 1. The table links selected foundational papers to their theoretical transition and possible clinical translation within a predictive vestibular framework.

## 21. Clinical Domains Suggested by a Predictive Vestibular Framework

The domain-based clinical translation of the predictive vestibular framework is summarized in Table 2. The table links each clinical domain to representative tests or observations and to its possible interpretation within a multidomain vestibular model.

## 22. Conclusions

Kathleen E. Cullen’s work provides one of the clearest conceptual routes from classical vestibular reflex physiology to a modern science of predictive self-motion control. The long lesson is that the vestibular system does not simply detect motion. It predicts, filters, integrates, and interprets self-motion in relation to action, body configuration, gravity, and behavioral context.

For clinical otoneurology, this lesson is not abstract. It directly addresses the daily problem of patients whose symptoms exceed what a single reflex measurement can explain. A preserved vHIT does not exclude impaired self-motion estimation. Normal peripheral responses do not exclude maladaptive visual dependence. A weak or asymmetric sustained rotational response may reveal temporal integration defects invisible to impulse testing. Persistent dizziness may reflect not only residual vestibular damage, but also abnormal prediction, sensory reweighting, and body-centered control.

The modern vestibular patient may therefore not be the patient without reflexes, but the patient whose brain can no longer predict, interpret, and trust self-motion. This formulation is not a rejection of reflex physiology; it is an extension of it into the domains of action, prediction, embodied behavior, and clinical meaning.

The future of vestibular medicine should therefore be domain-based and integrative. The clinician should ask not only whether the labyrinth is damaged, but whether the patient can use vestibular information to stabilize gaze, orient the body, tolerate visual motion, predict the sensory consequences of movement, sustain coherent motion estimates over time, and maintain a stable sense of self-motion in the world. In this sense, Cullen’s experimental physiology does not remain in the laboratory. It becomes a grammar for understanding the modern vestibular patient, while the clinical framework proposed here should be regarded as hypothesis-generating and open to prospective validation.

## Figures and Tables

**Table 1:** Foundational papers and conceptual transitions in predictive vestibular neuroscience.

Selected Work	Key Concept	Theoretical Transition	Clinical Translation
Angelaki & Cullen, 2008, Annual Review of Neuroscience	Vestibular signals are immediately embedded in multimodal computations involving canal-otolith integration, gravity, proprioception, corollary discharge, reference frames, navigation, and spatial orientation.	Transition from the vestibular system as a reflex arc to the vestibular system as a multimodal computational sense.	Provides the conceptual foundation for interpreting preserved reflexes with impaired self-motion perception, visual-vestibular integration, body-centered stability, or active/passive prediction.
Cullen, 2011, Current Opinion in Neurobiology	Self-motion is encoded by combining vestibular signals with optic flow, proprioception, and motor efference copy; behavioral context shapes the optimal computation.	Transition from vestibular reflex coding to context-dependent neural representations of self-motion.	Explains why symptoms may emerge in real-life contexts despite preserved isolated reflex measures: the disorder may involve self-motion inference, cue weighting, or context-dependent integration.
Brooks & Cullen, 2009	Rostral fastigial neurons integrate vestibular and proprioceptive inputs to estimate body motion in space.	Transition from head-centered sensory coding to body-centered self-motion estimation.	Explains why head-centered tests may not fully capture body-centered instability.
Cullen et al., 2011	Internal models suppress predictable vestibular reafference during active movement.	Transition from passive sensory detection to prediction of expected versus unexpected motion.	Supports clinical interpretation of dizziness as impaired prediction, cancellation, or mismatch.
Cullen, 2012	Vestibular processing is intrinsically multimodal and contributes to reflexes, posture, perception, and motor control.	Transition from VOR-centered physiology to multimodal self-motion encoding.	Encourages pattern-based interpretation beyond a single peripheral test.
Carriot, Jamali & Cullen, 2015	The system updates internal models and reweights sensory inputs when vestibular information becomes unreliable.	Transition from static deficit models to adaptive sensory reweighting.	Foundation for compensation, visual dependence, rehabilitation, and persistent dizziness.
Cullen, 2019, Nature Reviews Neuroscience	Natural self-motion requires vestibular processing for perception and action in behaviorally relevant contexts.	Transition from laboratory reflex paradigms to natural self-motion, perception, and action.	Supports interpretation of real-life dizziness in motion, navigation, complex visual scenes, and functional behavior.
Cullen & Zobeiri, 2021	Proprioception and vestibular signals cooperate during active self-motion.	Transition from passive laboratory motion to natural predictive behavior.	Explains normal passive tests with impairment in walking, turning, visual complexity, or dual task.
Cullen, 2023, Trends in Neurosciences	The vestibular cerebellum computes internal models of eye, head, body, and gravity-related orientation.	Transition from regional anatomy to computational cerebellar models.	Links cerebellar vestibular processing with velocity storage, verticality, sustained self-motion, and visual dependence.
Cullen, 2023, Handbook of Clinical Neurology	Vestibular nuclei and motor pathways integrate vestibular afferents with extra-vestibular and predictive signals.	Transition from relays to multimodal computational hubs.	Supports interpretation of VVOR/VORS, HIMP/SHIMP, VOR cancellation, compensation, and central integration disorders.
Peripheral afferent diversity (calyx, bouton, dimorphic endings; regular and irregular afferents)	Vestibular information is diversified through different hair-cell contacts, afferent endings, discharge regularity, and coding strategies.	Transition from a single vestibular output to parallel dynamic channels for motion encoding.	Supports interpretation of test dissociations across rapid/transient and slow/sustained or integrative vestibular domains.
Manzari, 2026, Audiology Research	Multiscale integration of acceleration and jerk sensing links Type I/Type II hair-cell specialization, calyx/bouton/dimorphic afferent architecture, regular/irregular discharge, organ mechanics, and clinical vestibular tests.	Transition from a simple frequency-based interpretation to a dynamic-domain model in which acceleration- and jerk-sensitive activation modes coexist across vestibular processing.	Supports the interpretation of test dissociations across vHIT, SVIN, VEMPs, calorics, rotational chair, and post-rotatory responses as differences between transient/jerk-rich and sustained/acceleration-dominated or integrative domains.

**Table 2:** Clinical domains suggested by a predictive vestibular framework.

Clinical Domain	Representative Tests or Observations	Possible Interpretation
Reflexive	vHIT/HIMP, SHIMP, calorics, rotational chair gain	Integrity or impairment of vestibulo-ocular reflex pathways across frequency and acceleration domains.
Perceptual	Motion sensitivity, dizziness, spatial disorientation, subjective verticality	Impaired self-motion estimation, orientation relative to gravity, or mismatch between expected and actual sensory inflow.
Postural	Stance, gait, darkness, uneven surfaces, falls, body sway	Impaired transformation from head motion to body-centered stability or maladaptive use of sensory references.
Visual-vestibular	VVOR, VORS, optokinetic responses, visual dependence	Abnormal integration or suppression of vestibular input in visually complex contexts.
Sustained/temporal	Sinusoidal rotation, post-rotatory responses, time constants, velocity storage behavior	Disturbed temporal integration of self-motion, low-frequency processing, or maladaptive sustained vestibular dynamics.
Predictive/adaptive	Active versus passive movement complaints, avoidance, poor rehabilitation tolerance	Defective internal models, reafference cancellation, or sensory reweighting.

## Data Availability

No new data were created or analyzed in this study.
